# Native myocardial T1 mapping in pulmonary hypertension: correlations with cardiac function and hemodynamics

**DOI:** 10.1007/s00330-016-4360-0

**Published:** 2016-04-27

**Authors:** Ursula Reiter, Gert Reiter, Gabor Kovacs, Gabriel Adelsmayr, Andreas Greiser, Horst Olschewski, Michael Fuchsjäger

**Affiliations:** 1 Division of General Radiology, Department of Radiology, Medical University of Graz, Auenbruggerplatz 9/P, A-8036 Graz, Austria; 2 Siemens Healthcare, Graz, Austria; 3 Division of Pulmology, Department of Internal Medicine, Medical University of Graz, & LBI for Lung Vascular Research, Graz, Austria; 4 Siemens Healthcare, Erlangen, Germany

**Keywords:** T1 mapping, Cardiac magnetic resonance imaging, Pulmonary hypertension, Right heart hemodynamics, Cardiac function

## Abstract

**Objectives:**

To analyze alterations in left ventricular (LV) myocardial T1 times in patients with pulmonary hypertension (PH) and to investigate their associations with ventricular function, mass, geometry and hemodynamics.

**Methods:**

Fifty-eight patients with suspected PH underwent right heart catheterization (RHC) and 3T cardiac magnetic resonance imaging. Ventricular function, geometry and mass were derived from cine real-time short-axis images. Myocardial T1 maps were acquired by a prototype modified Look-Locker inversion-recovery sequence in short-axis orientations. LV global, segmental and ventricular insertion point (VIP) T1 times were evaluated manually and corrected for blood T1.

**Results:**

Septal, lateral, global and VIP T1 times were significantly higher in PH than in non-PH subjects (septal, 1249 ± 58 ms vs. 1186 ± 33 ms, *p* < 0.0001; lateral, 1190 ± 45 ms vs. 1150 ± 33 ms, *p* = 0.0003; global, 1220 ± 52 ms vs. 1171 ± 29 ms, *p* < 0.0001; VIP, 1298 ± 78 ms vs. 1193 ± 31 ms, *p* < 0.0001). In PH, LV eccentricity index was the strongest linear predictor of VIP T1 (*r* = 0.72). Septal, lateral and global T1 showed strong correlations with VIP T1 (*r* = 0.81, *r* = 0.59 and *r* = 0.75, respectively).

**Conclusions:**

In patients with PH, T1 times in VIPs and in the entire LV myocardium are elevated. LV eccentricity strongly correlates with VIP T1 time, which in turn is strongly associated with T1 time changes in the entire LV myocardium.

***Key Points*:**

*• Native T1 mapping detects left ventricular myocardial alterations in pulmonary hypertension*

*• In pulmonary hypertension, native T1 times at ventricular insertion points are increased*

*• These T1 times correlate strongly with left ventricular eccentricity*

*• In pulmonary hypertension, global and segmental myocardial T1 times are increased*

*• Global, segmental and ventricular insertion point T1 times are strongly correlated*

## Introduction

Patients with pulmonary hypertension (PH) are at risk for progressive heart failure [1, 2]. The extent of late gadolinium enhancement at the ventricular insertion points (VIPs) and intraventricular septum (IVS) on cardiac magnetic resonance (CMR) imaging of these patients has been identified as a marker of progression and clinical worsening of disease [3–6]. As late gadolinium enhancement (LGE) imaging detects focal myocardial lesions but cannot assess diffuse myocardial remodelling [7], it remains unclear whether myocardial alterations in PH are restricted to the VIPs and IVS, or if the global left ventricular (LV) myocardium is affected by chronic right heart pressure overload [8, 9].

Myocardial T1 relaxation time mapping is an emerging technique for tissue characterization that provides information about tissue composition on a standardized scale [7, 10]. Increasing with myocardial collagen content, myocardial T1 times have the potential to identify both focal and diffuse myocardial fibrosis on global as well as regional levels [11–13]. We hypothesized that in patients with PH, T1 maps could depict myocardial alterations at the VIPs and IVS and, if present, in the entire LV myocardium.

The aim of the present study was, therefore, to assess LV global, segmental and VIP T1 times in patients with normal and elevated mean pulmonary arterial pressure, and to investigate their correlations with ventricular function, mass, geometry and hemodynamic parameters.

## Methods

### Study population

This prospective, explorative study was approved by the local ethical review board, and all subjects gave written informed consent. Patients with contraindications to magnetic resonance imaging were not enrolled.

From November 2012 to January 2015, a total of sixty-four patients with suspected PH underwent right heart catheterization (RHC) and non-contrast CMR imaging. Patients with known cardiomyopathies or PH owing to left heart diseases [1] were excluded from analysis. The remaining fifty-eight patients underwent both investigations within 7 ± 10 days. Demographic characteristics of the study population are given in Table 1.

**Table 1:** Demographic characteristics and RHC data of the study population. Parameters are given as means ± standard deviations. *p* values refer to comparison of mean values of non-PH and PH subjects. The PH groups are defined as follows: 1 - pulmonary arterial hypertension (PAH), 3 - PH due to lung diseases and/or hypoxia, 4 - chronic thromboembolic PH, and 5 - PH with unclear and/or multifactorial mechanisms

Parameter	All	Non-PH	PH	*p*
No. of patients	58	23	35	
PH group 1 patients			18	
PH group 3 patients			7	
PH group 4 patients			8	
PH group 5 patients			2	
No. of female/male patients	36/22	16/7	20/15	
age (years)	62 ± 15	59 ± 12	64 ± 16	0.23
BSA	1.8 ± 0.2	1.8 ± 0.2	1.8 ± 0.2	0.93
HF (beats/min)	72 ± 12	68 ± 11	74 ± 12	0.052
mBP (mmHg)	84 ± 12	87 ± 10	83 ± 13	0.27
sBP (mmHg)	125 ± 20	132 ± 21	120 ± 17	0.016
dBP (mmHg)	68 ± 12	69 ± 10	68 ± 14	0.71
mPAP (mmHg)	34 ± 17	18 ± 4	44 ± 13	<0.0001
sPAP (mmHg)	55 ± 26	29 ± 6	72 ± 19	<0.0001
dPAP (mmHg)	22 ± 12	11 ± 3	29 ± 11	<0.0001
PAWP (mmHg)	9 ± 3	8 ± 3	9 ± 3	0.090
RAP (mmHg)	7 ± 4	5 ± 2	8 ± 4	0.0011
PVR (Wood units)	6.3 ± 5.1	2.0 ± 1.1	9.1 ± 4.7	<0.0001

Twenty healthy subjects (10 women; mean age of all subjects, 33 ± 12 years; age range, 21–54 years) without any history of cardiovascular and/or pulmonary disease and with normal left and right ventricular cardiac function and myocardial mass [14] also underwent MR imaging so that the 3T myocardial T1 blood normalization coefficient could be determined [15].

### Right heart catheterization

RHC was performed in free breathing in the supine position with a 7F quadruple-lumen, balloon-tipped, flow-directed Swan-Ganz catheter (Baxter Healthcare Corp., Irvine, CA, USA) using the transjugular approach. The parameters obtained included mean, systolic and diastolic pulmonary arterial pressure (mPAP, sPAP and dPAP, respectively), pulmonary arterial wedge pressure (PAWP), right atrial pressure (RAP) and pulmonary vascular resistance (PVR) with the cardiac output measured by thermodilution. Moreover, mean, systolic and diastolic systemic blood pressure (mBP, sBP and dBP, respectively) were measured during RHC. RHC and systemic blood pressure parameters are summarized under the term hemodynamic parameters.

PH was diagnosed in thirty-five patients. Sixteen subjects with mPAP < 21 mmHg (mPAP = 16 ± 3 mmHg; range, 11–19 mmHg) and seven patients who demonstrated borderline mPAP (range, 21–24 mmHg) were labelled as non-PH subjects. The RHC-based clinical classifications and hemodynamic parameters of the study population are summarized in Table 1. mPAP of patients with pulmonary arterial hypertension (PAH; PH group 1) compared to mPAP of PH patients without PAH (non-PAH PH; PH groups 3–5) did not differ significantly (mPAP = 46 ± 17 mmHg vs. mPAP = 42 ± 8 mmHg, *p* = 0.35).

### CMR imaging

CMR imaging was performed on a 3 T MR scanner (Magnetom Trio, Siemens Healthcare, Erlangen, Germany) using a phased-array 6-channel body matrix coil together with a spine matrix coil. Subjects were investigated in the supine position. For functional assessment, ECG-gated balanced steady-state free-precession (bSSFP) cine real-time short-axis images covering the entire right and left ventricles from base to apex were obtained under free breathing. Typical imaging parameters were temporal resolution, 46–51 ms; echo time, 1.0 ms; flip angle, 50–60°; field of view (FOV), 248–315 × 360 mm^2^; voxel size, 4.1–4.8 × 2.8 × 8.0 mm^3^.

A single breath-hold, ECG-gated modified Look-Locker inversion recovery (MOLLI) prototype sequence with single-shot bSSFP readout, motion correction and automatic T1 map generation was used to acquire basal, mid-ventricular and apical short-axis myocardial T1 maps in systole [10, 16]. The MOLLI scheme consisted of the acquisition of a total of seven images. Five images were acquired after an initial non-slice-selective inversion pulse at an inversion time of TI = 90 ms to 4 × RR + 90 ms. After a recovery phase of 5 heartbeats, 2 further images were measured after a second non-slice-selective inversion pulse at TI = 170 ms and RR + 170 ms. Protocol parameters of the bSSFP readout were repetition time, 2.6 ms; echo time, 1.1 ms; flip angle, 35°; FOV, 315 × 360 mm^2^; voxel size, 2.1 × 1.4 × 8.0 mm^3^. Generalized auto-calibrating partially parallel acquisition (GRAPPA) with a parallel acquisition factor of 2 and partial Fourier 6/8 reconstruction were employed to minimize acquisition time within each cardiac interval.

### CMR image analysis

Quantification of LV and right ventricular (RV) end-diastolic volume (EDV), end-systolic volume (ESV), ejection fraction (EF), stroke volume (SV) and muscle mass (MM) was performed with Argus function software (Siemens Healthcare, Erlangen, Germany) by manual segmentation of myocardial end-diastolic and end-systolic epicardial and endocardial borders including papillary muscles and trabeculae to the myocardium. EDV, ESV, SV and MM were normalized to body surface area (BSA). Indexed quantities are indicated with “I”, and CI denotes the cardiac index. The ventricular mass ratio (VMR) was calculated as the ratio RVMM/LVMM.

The LV eccentricity index (LVEI) was obtained from an end-diastolic mid-chamber short-axis image as the ratio of the LV diameters parallel and perpendicular to the IVS [17]. Maximal LV curvature ratio (LVCR) was calculated from the ratio of interventricular septal to free-wall curvature on a basal short-axis image in the cardiac phase with maximum IVS displacement [18].

**Fig. 1:**
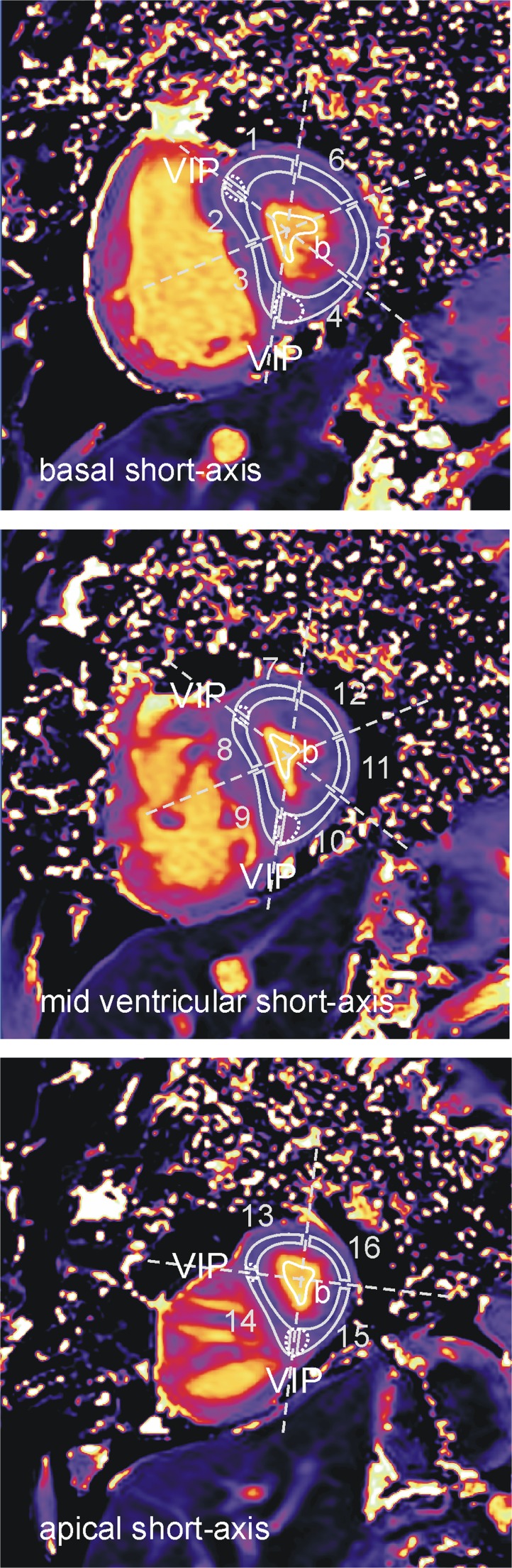
Segmentation of myocardial T1 maps. LV myocardium was segmented into 16 segments (solid gray contours) according to the AHA segmentation scheme (*gray dotted lines*). Regions at anterior septal and posterior septal ventricular insertions points (VIPs, *white dotted contours*) were drawn to be as large as possible around maximal T1 times in VIPs, targeting standard deviations (SDs) comparable with lateral myocardium. Blood T1 was derived from a region of interest (*b*, *white solid contour*) drawn in the blood pool of the left ventricular cavity

Two readers independently evaluated the T1 maps. Segmental LV myocardial T1 times were derived by manually outlining T1 maps according to the American Heart Association (AHA) segmentation scheme, carefully excluding blood pool, papillary muscles, trabeculae and epicardial structures (Fig. 1). Regions were drawn to be as large as possible while avoiding inclusion of subendocardial blood and subepicardial tissue boundaries. Regions of artificially high or low T1 values due to improper motion correction or partial volume effects were excluded from segmentation. Global LV myocardial T1 times were calculated as means of segmental values. Septal and lateral myocardial T1 times were calculated as means of septal (segments 2/3/8/9/14) and lateral (segments 5/6/11/12/16) myocardial segments. Regional VIP T1 times were defined as the mean T1 times of a region of maximum T1 values between the segments 1/2, 3/4, 7/8, 9/10, 13/14 and 14/15, respectively. The extent of each region was adapted by targeting its T1 time standard deviation (SD) to be comparable with the SDs in lateral myocardial segments (resulting in regions of typically 10–15 pixels). VIP T1 time was defined as the mean of anterior septal and posterior septal basal, mid-ventricular and apical short-axis VIP T1 times. Blood T1 time was derived from the mean of regions of interest drawn in the blood pool of the LV cavity in basal, mid-ventricular and apical short-axis slices. Regions were drawn to be as large as possible while avoiding subendocardial blood-tissue boundaries, papillary muscles and trabeculae.

All measured segmental, myocardial and VIP T1 times were normalized to the mean blood T1 time of the study population (T1_blood,mean_) assuming a common linear increase (k, blood normalization coefficient) of T1 times with the T1 of blood [15]. Given measured segmental, myocardial, VIP T1 and blood T1 (T1_blood_), blood - normalized segmental, myocardial and VIP T1 times were obtained from T1_normalized_ = T1 + k · (T1_blood,mean_ – T1_blood_), with k estimated from healthy subjects.

### Myocardial T1 blood normalization coefficient

Basal, mid-ventricular and apical short-axis T1 maps were acquired in 20 healthy subjects. The MOLLI protocol and the method of evaluating T1 maps used were the same as those described above. Blood normalization coefficient k was determined as the common slope of multiple linear regression of observer-averaged segmental T1 time with observer-averaged blood T1 time and AHA segments as a discrete independent variable.

### Statistical analysis

Mean values are given together with SDs; correlation coefficients are specified together with 95 % confidence intervals (CIs), if appropriate. Statistical analysis was performed using NCSS (Hintze, J. 2008, NCSS, LLC. Kaysville, UT, USA). A significance level of 0.05 was employed for statistical tests; no multiple comparison adjustment was applied.

The assumption of segment independence of the slope of multiple linear regression of observer-averaged segmental T1 time with observer-averaged blood T1 time and AHA segments in healthy volunteers was analyzed by including an interaction term in the model and testing the significance of this term via an F test. Segment dependence of intercepts of the multiple linear regression model was investigated by testing the significance of the AHA segment term of the model.

Interobserver variability for determination of T1 times in patients was calculated from paired measurements of the two readers as within-subject SD in variance component analysis and additionally as intraclass correlation coefficient r_IC_. Averages of T1 measurements of the two readers were used in subsequent analysis.

PH and non-PH patient group means as well as means of PAH and non-PAH PH patients were compared by a *t* test or Aspin–Welch unequal variance test if appropriate. Relationships between various T1 times and ventricular function, geometry, myocardial mass and hemodynamic parameters were investigated by correlation and linear regression analysis.

Moreover, the following multiple linear regression models were studied: The PH dependence of slopes and intercepts of linear regression models of septal, lateral and global myocardial T1 times on VIP T1 time were analyzed by adding the presence of PH as a discrete variable (with and without interaction) to the corresponding linear regression models and afterwards testing the significance of these additionally included terms. Bilinear regressions of VIP T1 time with LVEI and the other ventricular function, geometry, myocardial mass and hemodynamic parameters were studied for PH patients to analyze if one of the additional parameters can explain a significant amount of residual variation in VIP T1 time. Finally, all bilinear and trilinear regression models of septal, lateral and global myocardial T1 times with ventricular function, geometry, myocardial mass or hemodynamic parameters were analyzed for all patients and PH patients only to investigate if one of the independent parameter combinations can explain variations of septal, lateral and global myocardial T1 times similar to VIP T1 time.

## Results

### Ventricular function, mass and geometry indices

Ventricular function, mass and geometrical parameters were evaluated in all subjects and compared between patient groups. Table 2 summarizes the results of the comparisons as well as the correlations between the measured parameters and mPAP.

**Table 2:** Ventricular function, mass and geometry indices and their correlations with mPAP. *p* values describe the significance level of the difference in mean values of non-PH and PH subjects

Parameter	All	r	Non-PH	PH	*p*
RVEDVI	109 ± 40	0.28	91 ± 23	122 ± 44	0.0010
RVESVI	62 ± 37	0.43	43 ± 15	75 ± 41	<0.0001
RVSVI	47 ± 14	-0.35	48 ± 10	46 ± 15	0.62
RVEF	46 ± 13	-0.63	54 ± 7	41 ± 14	<0.0001
RVCI	3.3 ± 1.0	-0.14	3.2 ± 0.9	3.4 ± 1.1	0.44
RVMMI	46 ± 22	0.63	31 ± 8	56 ± 22	<0.0001
LVEDVI	68 ± 19	-0.17	71 ± 17	66 ± 20	0.34
LVESVI	24 ± 12	0.09	24 ± 10	25 ± 14	0.69
LVSVI	43 ± 12	-0.37	47 ± 10	41 ± 12	0.045
LVEF	65 ± 10	-0.29	67 ± 7	63 ± 11	0.11
LVCI	3.0 ± 0.6	-0.23	3.1 ± 0.6	3.0 ± 0.7	0.47
LVMMI	52 ± 13	0.06	51 ± 17	53 ± 11	0.64
VMR	0.90 ± 0.34	0.65	0.65 ± 0.22	1.06 ± 0.31	<0.0001
LVEI	1.19 ± 0.19	0.76	1.07 ± 0.07	1.27 ± 0.19	<0.0001
LVCR	0.16 ± 0.67	-0.87	0.79 ± 0.17	-0.26 ± 0.52	<0.0001

### Myocardial T1 blood normalization coefficient

All myocardial segments (*n* = 320) in T1 maps from the 20 healthy subjects were evaluable. Multiple linear regression of segmental myocardial T1 values with blood T1 time and AHA segments revealed multiple correlation coefficient *R* = 0.66. Intercepts differed significantly for segments (*p* < 0.0001), whereas slopes did not (*p* = 0.071). The common slope *k* = 0.30 was employed as the blood normalization coefficient in the study population.

### Blood T1 time

Interobserver variability for the determination of blood T1 time was 10 ms (*r*_*IC*_ = 0.99). Mean blood T1 times of 1890 ± 91 ms for non-PH and 1822 ± 101 ms for PH subjects differed (*p* = 0.011). Myocardial T1 times were normalized to the study population’s mean blood T1 time of 1849 ± 102 ms.

### Segmental, septal, lateral and global myocardial T1 times

T1 times were evaluated in 927 of 928 myocardial segments. One segment (0.1 %) was excluded from analysis due to close proximity to the LV outflow tract. Interobserver variability levels for determination of segmental, septal, lateral and global myocardial T1 values were 15 ms (*r*_*IC*_ = 0.96), 8 ms (*r*_*IC*_ = 0.98), 9 ms (*r*_*IC*_ = 0.97) and 7 ms (*r*_*IC*_ = 0.98), respectively.

**Fig. 2:**
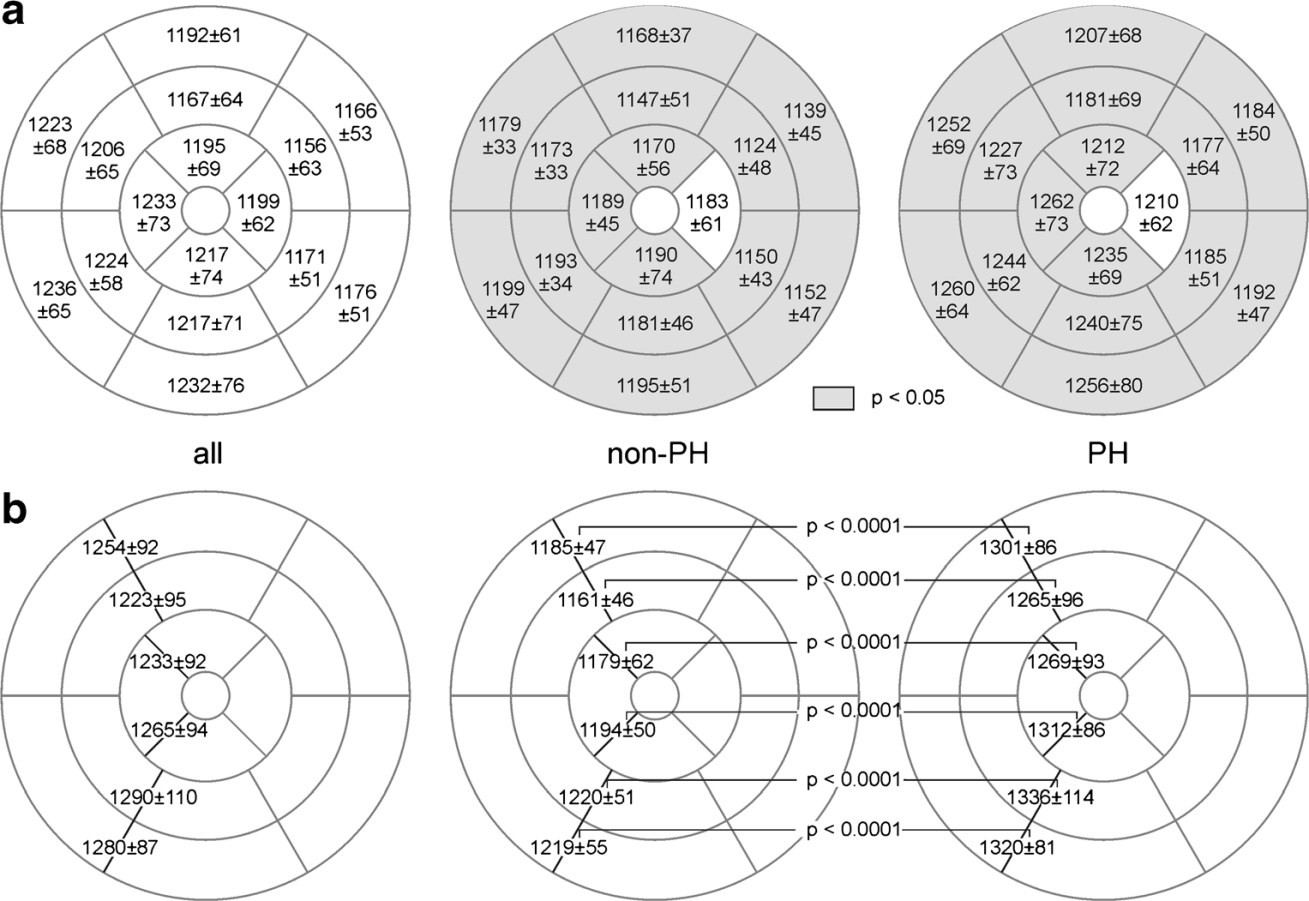
Mean segmental myocardial and regional VIP T1 times for all, non-PH and PH patients. Mean values and standard deviations (in ms) of segmental myocardial (a) and regional VIP (b) T1 times are specified in bull’s-eye plots. Segments for which T1 values differed significantly between patients with and without PH are in *gray*; *p* values for the comparison of regional VIP T1 times are indicated

**Fig. 3:**
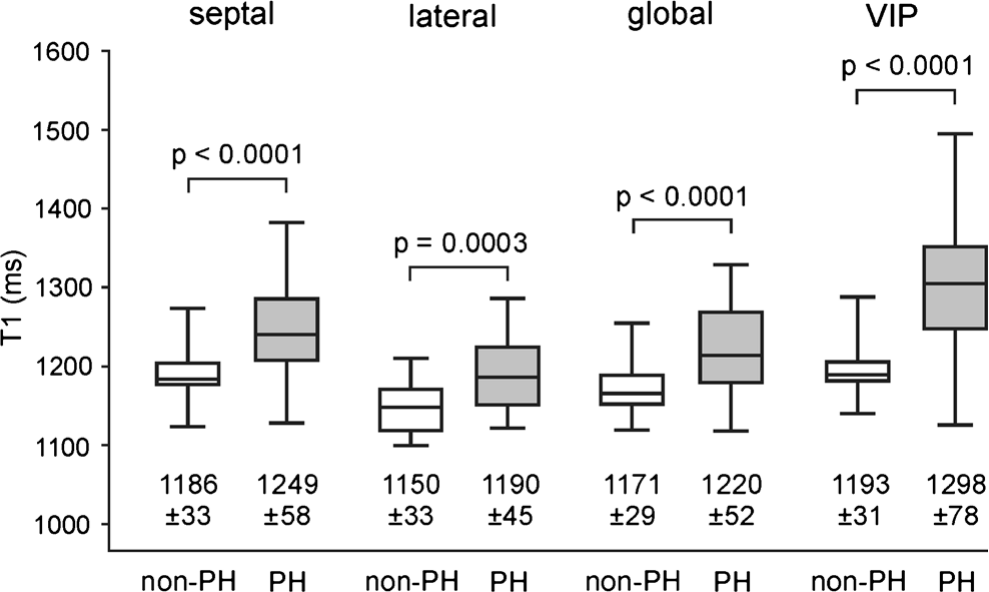
Box plot of septal, lateral, global and VIP T1 values in patients with and without PH. Mean values and standard deviations are specified in ms; *p* values are given above brackets for the corresponding comparisons of non-PH and PH subjects

Mean segmental myocardial T1 times are given in Fig. 2a. In all segments, T1 values were higher in patients with PH than in those without PH; the difference was significant in 15 segments. Mean septal, lateral and global myocardial T1 times were significantly higher in patients with PH (Fig. 3). Neither segmental, septal, lateral nor global myocardial T1 times differed significantly between PAH and non-PAH PH groups. Moreover, septal, lateral and global T1 times correlated strongly with each other: *r* = 0.94 (global vs. septal; 95 % CI 0.91–0.97), *r* = 0.92 (global vs. lateral; 95 % CI 0.87–0.95), and *r* = 0.79 (septal vs. lateral; 95 % CI 0.67–0.87).

### VIP T1 times

Three of the 348 regional VIP T1 times (0.9 %) were rated as non-evaluable and excluded from the analysis: 2 VIPs due to artefacts caused by incorrect motion correction and 1 VIP due to close proximity to the LV outflow tract. Interobserver variability levels for the assessment of regional and (averaged) VIP T1 times were 25 ms (*r*_*IC*_ = 0.93) and 14 ms (*r*_*IC*_ = 0.97), respectively.

T1 times of anterior septal and posterior septal basal, mid-ventricular and apical VIPs were significantly higher in patients with PH than in those without PH (Fig. 2b). The mean VIP T1 time of the PH group was significantly higher than that of the non-PH group (Fig. 3). No significant differences were found in VIP T1 times of PAH and non-PAH PH patients.

**Fig. 4:**
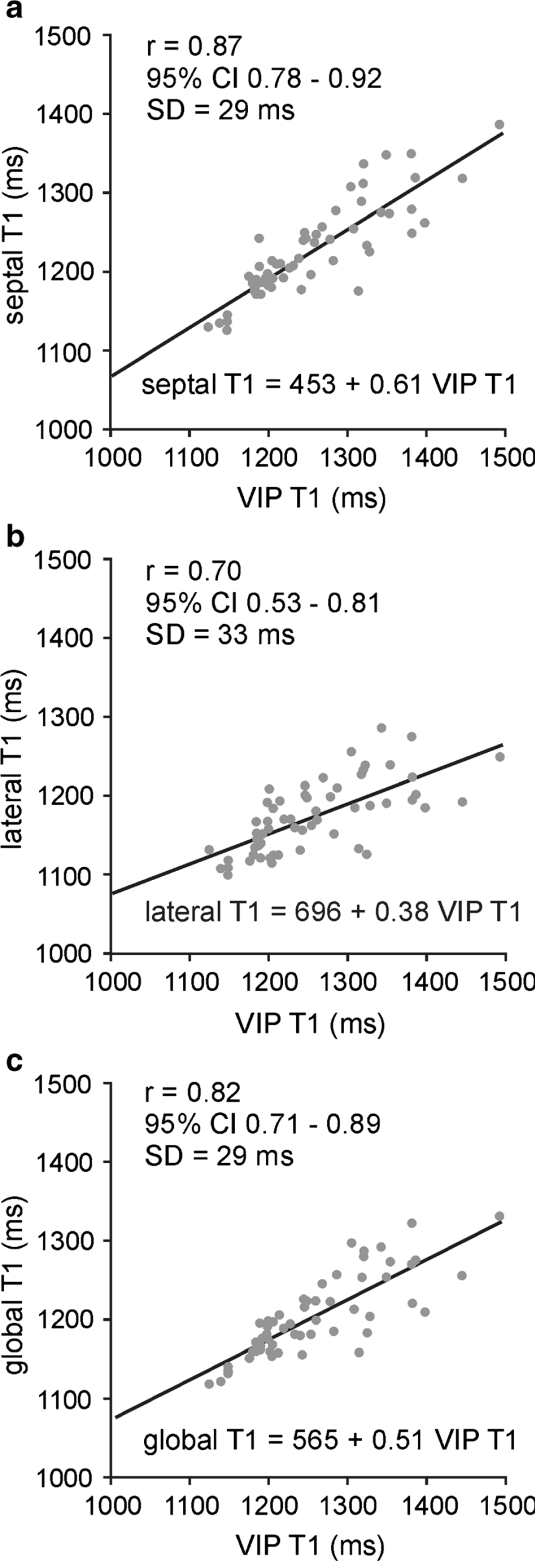
Linear regression of septal (a), lateral (b) and global (c) myocardial T1 times on VIP T1 time in all subjects. Correlation coefficient r is given together with 95 % confidence intervals (CIs) in parentheses; SD denotes the standard deviation of residuals from the regression line

VIP T1 values correlated with septal, lateral and global myocardial T1 times. Correlation coefficients and linear regression lines for all patients are given in Fig. 4. Neither slopes nor intercepts of the regression lines depended significantly on the presence of PH. Correlation coefficients of VIP T1 time with septal, lateral and global myocardial T1 times for PH patients were *r* = 0.81 (95 % CI 0.64–0.90), *r* = 0.59 (95 % CI 0.32–0.77) and *r* = 0.75 (95 % CI 0.54–0.86), respectively.

### Correlations of T1 times with hemodynamic, ventricular function, mass and geometrical parameters

Correlations of septal, lateral, global and VIP T1 times with hemodynamic, ventricular function, mass and geometrical parameters for all patients and PH patients are summarized in Table 3.

**Table 3:** Correlation of septal, lateral, global and VIP T1 times with hemodynamic, ventricular function, mass and geometrical parameters for all and PH patients. Values are correlation coefficients

Parameter	VIP T1		Septal T1		Lateral T1		Global T1	
	All	PH	All	PH	All	PH	All	PH
mPAP	0.78	0.62	0.58	0.34	0.42	0.13	0.50	0.23
sPAP	0.73	0.50	0.53	0.20	0.38	0.01	0.45	0.08
dPAP	0.80	0.67	0.62	0.43	0.47	0.25	0.55	0.34
PAWP	0.12	-0.07	0.16	-0.01	0.09	-0.11	0.12	-0.06
RAP	0.39	0.25	0.32	0.19	0.21	0.03	0.30	0.16
PVR	0.75	0.59	0.53	0.32	0.40	0.15	0.47	0.23
mBP	-0.12	-0.01	-0.09	0.00	-0.10	-0.06	-0.10	-0.05
sBP	-0.43	-0.43	-0.31	-0.32	-0.29	-0.27	-0.31	-0.33
dBP	0.00	0.10	0.00	0.08	-0.01	0.02	0.00	0.05
RVEDVI	0.31	0.14	0.41	0.33	0.27	0.22	0.35	0.27
RVESVI	0.48	0.32	0.53	0.44	0.36	0.29	0.46	0.37
RVSVI	-0.37	-0.46	-0.24	-0.25	-0.20	-0.16	-0.23	-0.23
RVEF	-0.63	-0.54	-0.57	-0.47	-0.38	-0.29	-0.50	-0.40
RVCI	-0.17	-0.35	-0.08	-0.13	-0.03	-0.07	-0.06	-0.10
RVMMI	0.68	0.55	0.68	0.62	0.48	0.41	0.59	0.51
LVEDVI	-0.10	-0.03	0.04	0.08	-0.06	0.00	-0.05	-0.01
LVESVI	0.17	0.22	0.27	0.31	0.18	0.24	0.20	0.26
LVSVI	-0.34	-0.30	-0.22	-0.22	-0.29	-0.27	-0.30	-0.30
LVEF	-0.37	-0.37	-0.39	-0.40	-0.30	-0.31	-0.35	-0.38
LVCI	-0.20	-0.26	-0.09	-0.12	-0.15	-0.20	-0.16	-0.19
LVMMI	0.22	0.42	0.36	0.53	0.21	0.34	0.28	0.45
VMR	0.60	0.44	0.51	0.43	0.38	0.29	0.46	0.35
LVEI	0.79	0.72	0.67	0.60	0.49	0.38	0.62	0.54
LVCR	-0.75	-0.56	-0.65	-0.51	-0.50	-0.35	-0.58	-0.42

**Fig. 5:**
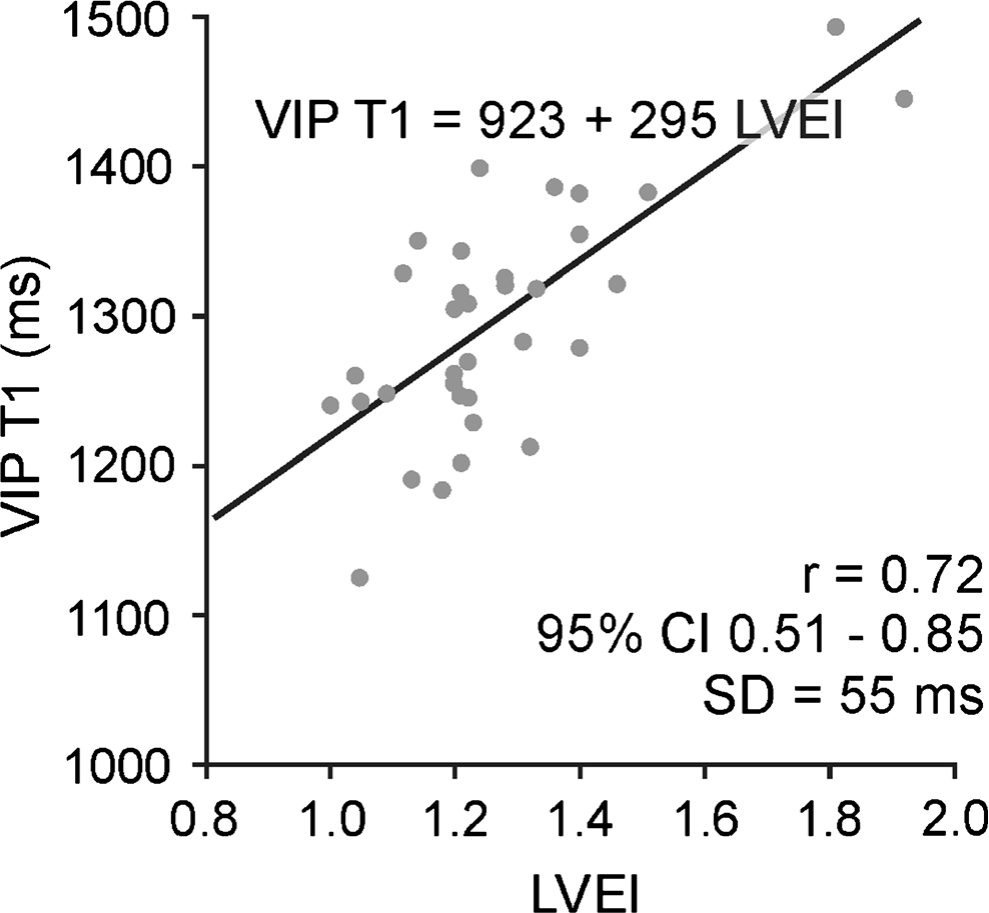
Linear regression of VIP T1 time on LVEI in patients with PH. Correlation coefficient r is given together with 95 % confidence intervals (CIs) in parentheses; SD denotes the standard deviation of residuals from the regression line

LVEI was the strongest correlate of VIP T1 time for patients with PH; the corresponding linear regression of VIP T1 time on LVEI is shown in Fig. 5. The only parameter reaching significance additional to LVEI in bilinear regression was RVCI: The multiple correlation coefficient was *R* = 0.76 with partial correlation coefficients of *r* = 0.72 (*p* < 0.0001) and *r* = -0.34 (*p* = 0.049) for LVEI and RVCI, respectively.

Moreover, there was no bilinear or trilinear combination of hemodynamic, ventricular function, mass or geometrical parameters, which correlated stronger with septal, lateral or global myocardial T1 times than VIP T1 time, both for all patients and PH patients.

### Impact of blood normalization

Non-normalized T1 values in all segments and of anterior septal and posterior septal basal, mid-ventricular and apical VIPs were higher in patients with PH than in those without PH. Whereas the difference remained significant for all VIPs, the differences of segmental T1 times were significant only in seven segments (2–4, 8–10, 14).

**Fig. 6:**
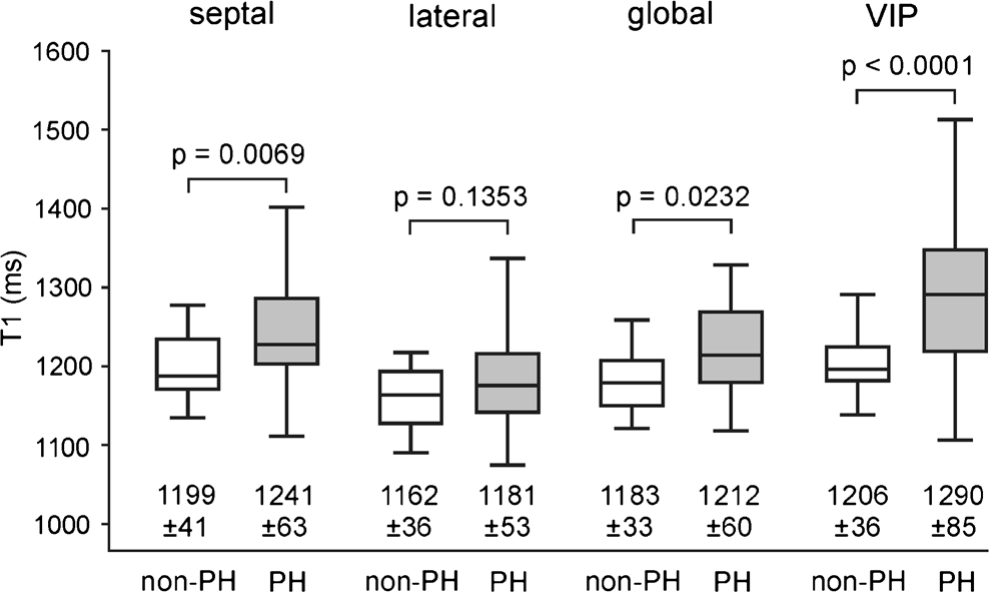
Box plot of non-normalized septal, lateral, global and VIP T1 values in patients with and without PH. Mean values and standard deviations are specified in ms; *p* values are given above brackets for the corresponding comparisons of non-PH and PH subjects

Accordingly, mean septal, lateral and global non-normalized myocardial T1 times as well as mean non-normalized VIP T1 time were higher in patients with PH than in those without PH. The difference for the lateral wall, however, was not significant (Fig. 6).

Correlation coefficients of non-normalized VIP T1 time with septal, lateral and global non-normalized myocardial T1 times remained similar to the blood normalized values [all patients, *r* = 0.86 (95 % CI 0.77–0.91), *r* = 0.67 (95 % CI 0.50–0.79), and *r* = 0.80 (95 % CI 0.68–0.88), respectively; PH patients, *r* = 0.84 (95 % CI 0.70–0.92), *r* = 0.67 (95 % CI 0.43–0.82), and *r* = 0.79 (95 % CI 0.61–0.89), respectively]. LVEI remained the strongest correlate of non-normalized VIP T1 time for patients with PH (*r* = 0.65, 95 % CI 0.39–0.80).

## Discussion

The major findings of the present study were that 1) septal, lateral, global and VIP T1 times were significantly higher in patients with PH than in those without PH; 2) VIP T1 time was the strongest linear predictor of septal, lateral and global T1 times; and 3) in patients with PH, LVEI was the strongest linear predictor of VIP T1 time, and RVCI was the only additional significant parameter in bilinear regression.

As a consequence of ventricular interdependence, PH impairs both RV and LV function [19–24]: Progressive RV dilatation shifts the IVS toward the LV, successively altering LV geometry and contractility [17, 18, 25–29]. LV deformation and paradoxical IVS motion have been shown to lead to high levels of stress at VIPs [5, 30], which in turn affect the regional architecture of myocardial tissue [4, 23, 31, 32]. Disarrayed myocardium containing plexiform fibrosis, where collagen volume is increased between myocardial fibres but does not replace them, has been reported from autopsies of PH patients [4, 32]. In a recent experimental large-animal model study of chronic PH [33], native T1 values at the VIPs have been demonstrated to be increased due to increased interstitial collagen associated with fibre disarray.

Accordingly, we presume that the significantly higher VIP T1 times found in patients with PH can be explained by focal myocardial remodelling in all (anterior and posterior) VIPs at the basal, mid-ventricular and apical levels.

To lessen the impact of interobserver variability, analysis was restricted to mean VIP T1 times. In agreement with relationships previously reported between LGE volume and hemodynamic, function, mass and geometry parameters [3–5, 29], univariate analysis of VIP T1 times revealed various significant correlations with these parameters. VIP T1 time correlated most strongly with LVEI in patients with PH. Interpreting the magnitude of VIP T1 as a measure of progression of myocardial remodelling comparable with the extent of LGE at VIPs, this result is in accordance with findings of Sato et al. [29], who identified paradoxical IVS motion as the sole predictor of LGE at VIPs. The appearance of RVCI as an additional predictive variable in PH patients might indicate the interrelationship between myocardial remodelling at the VIPs and progression of right heart failure under the condition of right heart pressure overload [4, 6]. Furthermore, no differences in VIP T1 times were found between PAH and non-PAH PH patients, which is in accordance with the aetiology independence of LGE at VIPs observed previously [29, 32, 34].

In our study, alterations of LV myocardial T1 times in patients with PH were not limited to the VIPs and IVS. T1 times were higher in PH patients in all LV segments, whereas significance was reached for all but the apical lateral segment. Segmental averaging reduced variances of determined T1 times, such that septal, global and lateral T1 values were all significantly higher in patients with PH than in patients without PH. No differences were found between PAH and non-PAH PH patients. We conjecture that the presence of higher T1 times throughout the LV myocardium in patients with PH could be related to interstitial fibrosis; in turn, such fibrosis could explain the increased LV wall stiffness, the reduced LV longitudinal contractility and the reduced circumferential strain even at LV non-septal segments reported in patients with chronic PH [17, 20, 21].

The current study cannot unmask the underlying mechanisms leading to LV tissue alterations. However, given that VIP T1 time was strongly correlated with septal, global and lateral T1 times and no simple multilinear model of hemodynamic, ventricular function, mass and geometrical parameters could similarly predict these T1 values, it appears that PH involves the total LV myocardium to a large extent in parallel. This is compatible with the fact that VIP, septal, global and lateral T1 times showed similar significances in univariate correlations with hemodynamic, ventricular function, mass and geometrical parameters. Especially, significant correlations of lateral T1 times with RVMMI, RVEDVI, RVESVI and RVEF can be understood in this context, as these RV parameters are related to mPAP and PH. Moreover, consistently, neither PAWP as a surrogate for left atrial pressure, nor LV functional parameters exhibited dominant association with septal, global or lateral T1 times similar to the association of these parameters with VIP T1 times.

Segmental, myocardial and VIP T1 times were normalized to the mean blood T1 time of the study population. The blood normalization coefficient *k* = 0.30 derived from healthy volunteers at 3T was expectedly different from the one at 1.5T [15]. Blood normalization by trend did not change group differences or correlations. However, blood normalization decreased variances of T1 times in the study population as well as in PH and non-PH subgroups and, moreover, enhanced differences in mean T1 times between PH and non-PH groups by eliminating differences in blood T1 times. The impact was largest for lateral T1 times: Whereas only by trend higher in PH compared to non-PH subjects, differences became highly significant after blood normalization.

## Study limitations

The present study had a number of limitations. A non-contrast CMR protocol was applied, and, therefore, LGE and post-contrast T1 times could not be assessed. Analysis of LGE volume would have allowed proving the association of VIP T1 with the extent of LGE at VIPs. Post-contrast T1 and calculation of ECV might have provided additional information on the underlying nature of tissue alterations observed in native T1, as a recent pre-clinical study [33] suggests elevation of both native T1 and ECV in PH. Histological evaluation of the myocardium was lacking. Moreover, because reconstructed T1 maps were not applicable for evaluation of RV T1 times, RV myocardium could not be analyzed. Myocardial T1 times were blood-normalized based on a blood normalization coefficient determined from a healthy but not age-matched group. Blood normalization coefficient might vary with age; however, this would lead only to some minor remaining impact of blood T1 time in the results. Finally, the studied population was restricted to pre-capillary PH, but consisted of clinical PH groups of different sizes. Comparison of PAH and non-PAH PH patients did not reveal significant differences in T1 times. However, larger-scale studies are needed to investigate possible aetiology dependence of LV myocardial remodelling based on native T1 time alterations.

## Conclusions

In patients with PH, T1 times in VIPs and also in the entire LV myocardium are elevated. LV eccentricity strongly correlates with VIP T1 time, which in turn is strongly associated with T1 time changes in the entire LV myocardium. Further research is needed to determine if elevated LV T1 times have prognostic importance in patients with PH.

## Abbreviations

bSSFP: Balanced steady-state free-precession
BSA: Body surface area
CI: Confidence interval
CMR: Cardiac magnetic resonance
CO: Cardiac output
dBP: Diastolic systemic blood pressure
dPAP: Diastolic pulmonary arterial pressure
ECG: Electrocardiographically
EDVI: End-diastolic volume index
ESVI: End-systolic volume index
EF: Ejection fraction
FOV: Field of view
GRAPPA: Generalized auto-calibrating partially parallel acquisition
IVS: Intraventricular septum
K: Myocardial blood normalization coefficient
LGE: Late gadolinium enhancement
LV: Left ventricle
LVEI: Left ventricular eccentricity index
LVMMI: Left ventricular muscle mass index
LVCR: Left ventricular curvature ratio
mBP: Mean systemic blood pressure
MOLLI: Modified Look-Locker inversion recovery
mPAP: Mean pulmonary arterial pressure
PAH: Pulmonary arterial hypertension
PH: Pulmonary hypertension
PAWP: Pulmonary arterial wedge pressure
PVR: Pulmonary vascular resistance
RAP: Right atrial pressure
RHC: Right heart catheter
RV: Right ventricle
RVMMI: Right ventricular muscle mass index
sBP: Systolic systemic blood pressure
sPAP: Systolic pulmonary arterial pressure
SVI: Stroke volume index
TI: Inversion time
VIP: Ventricular insertion points
VMR: Ventricular mass ratio
